# Assessment of the medicinal potentials of the methanol extracts of the leaves and stems of *Buddleja saligna*

**DOI:** 10.1186/1472-6882-9-21

**Published:** 2009-07-06

**Authors:** Adeolu A Adedapo, Florence O Jimoh, Srinivas Koduru, Patrick J Masika, Anthony J Afolayan

**Affiliations:** 1 Department of Veterinary Physiology, Biochemistry and Pharmacology, University of Ibadan, Nigeria; 2 Department of Botany, University of Fort Hare, Alice 5700, South Africa; 3 ARDRI, University of Fort Hare, Alice 5700, South Africa

## Abstract

**Background:**

*Buddleja saligna *Willd (Loganiaceae) is a small to medium-sized evergreen tree; trunk short, often gnarled and crooked; crown dense, rounded or domed-shaped; foliage greyish green. The wild olives are traditionally used to lower blood pressures in many parts of the world. In southern Africa, bark and leaf decoctions are used to treat colic, coughs, colds, sore eyes, urinary problems and as purgatives.

**Methods:**

The antibacterial, antioxidant activities and phenolic contents of the methanol extracts of the leaves and stems of *Buddleja saligna *were evaluated using *in vitro *standard methods. Spectrophotometry was the basis for the determinations of total phenol, total flavonoids, flavonols, and proanthocyanidins. Tannins, quercetin and catechin equivalents were used for these parameters. The antioxidant activities of the leaves and stem extracts of *Buddleja saligna *were determined by ABTS, DPPH, and ferrous reducing antioxidant property (FRAP) methods. Laboratory isolates of 10 bacteria species which included five Gram-positive and five Gram-negative strains were used to assay for antibacterial activity of this plant.

**Results:**

The antioxidant activities of the leaves as determined by the ABTS and DPPH were similar to that of the stem. The flavonoids and the flavonols contents of the leaves were higher than that of the stem but the total phenols, proanthocyanidins and FRAP activities were higher in the methanol extracts of the stem. The extracts did show activity against both Gram-positive and Gram-negative bacteria. For instance, while the methanol extract of the leaves showed good activities on all the organisms except *Serratia marcescens *and *Pseudomonas aeruginosa *at MICs of between 2.5 and 5.0 mg/ml, the extract of the stem only showed activities on *Bacillus cereus, Streptococcus pyrogens *and *Pseudomonas aeruginosa *at the same concentration.

**Conclusion:**

The results from this study indicate that the leaves and stem extracts of *Buddleja saligna *possess antioxidant properties and could serve as free radical inhibitors or scavenger or, acting possibly as primary antioxidants. Although, the antibacterial properties of *Buddleja saligna *are not as effective as the standard drugs-Chloramphenicol and Streptomycin, they still possess some activity against bacterial strains used in this study. *Buddleja saligna *may therefore be a good candidate for functional foods as well as pharmaceutical plant-based products.

## Background

*Buddleja saligna *Willd (Loganiaceae) is a small to medium-sized evergreen tree; trunk short, often gnarled and crooked; crown dense, rounded or domed-shaped; foliage greyish green [1]. The plant is found usually on rocky hillsides or along watercourses. Fruits are edible but not tasty. Leaves are browsed by game and livestock. The wild olives are traditionally used to lower blood pressures in many parts of the world. In southern Africa, bark and leaf decoctions are used to treat colic, coughs, colds, sore eyes, urinary problems and as purgatives [1-3]. The dichloromethane extract of the leaves of this plant has shown some antimutagenic property [3]. No formal pharmacological studies are documented for this species. Many *Buddleja *species are however used as traditional medicines, in China and elsewhere. The results of an *in vitro *study [4]showed that an aqueous extract of one of these, *B. globosa*, used traditionally in the form of a poultice or lotion to promote wound healing, was able to stimulate the growth of dermal fibroblasts and reduce inflammation (two aspects of the cascade of events associated with the wound healing process). Activity in the study was attributed to flavonoids (luteolin, 6-hydroxyluteolin) and phenylethanoids (verbascoside, echinacoside) isolated from the aerial parts of *B. globosa*. Investigation of the secondary chemistry and bioactivity of *B. saligna *may demonstrate a similar mode of action. In a review of the bioactivity of *Buddleja *species [5], anti-inflammatory, antifungal and wound-healing properties are noted for members of the genus used in traditional medical practice.

Recent developments in biomedicals point to the involvement of free radicals in many diseases [6]. Free radicals attack the unsaturated fatty acids in the biomembranes resulting in membrane lipid peroxidation, a decrease in membrane fluidity, loss of enzymes, receptor activity and damage to membrane proteins leading to cell inactivation [7-9]. For these reasons antioxidants are of interest for the treatment of many kinds of cellular degeneration [10]. Antioxidants are compounds that inhibit or delay the oxidation process by blocking the initiation or propagation of oxidizing chain reactions [9].

It has long been recognized that naturally occurring substances in higher plants have antioxidant activity. There is a growing interest in oxygen-containing free radicals in biological systems and their implied roles as causative agents in the aetiology of a variety of chronic disorders. The harmful action of the free radicals can, however be blocked by antioxidant substances, which scavenge the free radicals and detoxify the organism. Current research into free radicals has confirmed that foods rich in antioxidants play an essential role in the prevention of cardiovascular diseases and cancers [10-12] and neurodegenerative diseases, including Parkinson's and Alzheimer's diseases [13], as well as inflammation and problems caused by cell and cutaneous aging [7,14].

Accordingly attention is focused on the protective biochemical functions of naturally occurring antioxidants in the cells of the organisms containing them [15-17]. Restriction is being imposed on the use of synthetic antioxidants because of their carcinogenicity, the need for natural antioxidants therefore become imperative and desirable [6,18,19]. Therefore as sources of natural antioxidants much attention is being paid to plants and other organisms. The antioxidant activity of phenols and other compounds present in oils have been well and widely studied by several authors [17,20-22].

Also, natural products from microorganisms have been the primary source of antibiotics, but with the increasing acceptance of herbal medicine as an alternative form of health care, the screening of medicinal plants for active compounds has become very important because these may serve as promising sources of novel antibiotic prototypes [23-25]. It has been shown that *in vitro *screening methods could provide the needed preliminary observations necessary to select crude plant extracts with potentially useful properties for further chemical and pharmacological investigations [26].

In the present study, the methanol extracts of the leaves and stem of *Buddleja saligna *were screened for antioxidant and antibacterial properties using standard procedures.

## Methods

### Plant collection

The plants were collected in July 2006 from some villages in the Eastern Cape Province of South Africa. The area falls within the latitudes 30°00–34° 15'S and longitudes 22° 45' – 30° 15'E. It is bounded by the sea in the east and the drier Karoo (semi-desert vegetation) in the west [27]. These areas consist of villages which are generally classified as rural and poor. The plants were identified by their vernacular names and later validated at the Department of Botany, University of Fort Hare and voucher specimens (Aded Med 2007/1–10) were deposited in the Griffen Herbarium of the University.

### Extract preparation

Plants were air dried at room temperature to get consistent weight. The dried plants were later ground to powder. Two hundred grams of ground plant material were shaken separately in methanol for 48 hrs on an orbital shaker. Extracts were filtered using a Buckner funnel and Whatman No 1 filter paper. Each filtrate was concentrated to dryness under reduced pressure at 40°C using a rotary evaporator. Each extract was resuspended in the respective solvent, methanol, to yield a 50 mg/ml stock solution [28].

### Chemicals

1,1-Diphenyl-2-picrylhydrazyl (DPPH), 2,2'-azinobis-3-ethylbenzothiazoline-6-sulfonic acid (ABTS), 3-(2-pyridyl)-5,6-diphenyl-1,2,4-triazine-4',4"-disulfonic acid, potassium ferricyanide; catechin, butylated hydroxytoluene (BHT), ascorbic acid, catechin, tannic acid, quercetin and FeCl_3 _were purchased from Sigma Chemical Co. (St. Louis, MO, USA)., vanillin from BDH; Folin-Ciocalteus's phenol reagent and sodium carbonate were from Merck Chemical Supplies (Damstadt, Germany). All the chemicals used including the solvents, were of analytical grade.

### Determination of total phenolics

Total phenol contents in the extracts were determined by the modified Folin-Ciocalteu method [29]. An aliquot of the extract was mixed with 5 ml Folin-Ciocalteu reagent (previously diluted with water 1:10 v/v) and 4 ml (75 g/l) of sodium carbonate. The tubes were vortexed for 15 sec and allowed to stand for 30 min at 40°C for color development. Absorbance was then measured at 765 nm using the Hewlett Packard UV-VS spectrophotometer. Samples of extract were evaluated at a final concentration of 0.1 mg/ml. Total phenolic content were expressed as mg/g tannic acid equivalent using the following equation based on the calibration curve: y = 0.1216x, R^2 ^= 0.9365, where x was the absorbance and y was the tannic acid equivalent (mg/g).

### Determination of total Flavonoids

Total flavonoids were estimated using the method of Ordon ez et al. [30]. To 0.5 ml of sample, 0.5 ml of 2% AlCl_3 _ethanol solution was added. After one hour at room temperature, the absorbance was measured at 420 nm. A yellow color indicated the presence of flavonoids. Extract samples were evaluated at a final concentration of 0.1 mg/ml. Total flavonoid content were calculated as quercetin (mg/g) using the following equation based on the calibration curve: y = 0.0255x, R^2 ^= 0.9812, where x was the absorbance and was the quercetin equivalent (mg/g).

### Determination of total Flavonols

Total flavonols in the plant extracts were estimated using the method of Kumaran and Karunakaran [14]. To 2.0 mL of sample (standard), 2.0 mL of 2% AlCl_3 _ethanol and 3.0 mL (50 g/L) sodium acetate solutions were added. The absorption at 440 nm was read after 2.5 h at 20°C. Extract samples were evaluated at a final concentration of 0.1 mg/ml. Total flavonoid content was calculated as quercetin (mg/g) using the following equation based on the calibration curve: y = 0.0255x, R^2 ^= 0.9812, where x was the absorbance and was the quercetin equivalent (mg/g).

### Determination of total proanthocyanidins

Determination of proanthocyanidin was based on the procedure reported by Sun et al. [31]. A volume of 0.5 ml of 0.1 mg/ml of extract solution was mixed with 3 ml of 4% vanillin-methanol solution and 1.5 ml hydrochloric acid; the mixture was allowed to stand for 15 min. The absorbance was measured at 500 nm. Extract samples were evaluated at a final concentration of 0.1 mg/ml. Total proanthocyanidin content were expressed as catechin equivalents (mg/g) using the following equation based on the calibration curve: y = 0.5825x, R^2 ^= 0.9277, where x was the absorbance and y is the catechin equivalent (mg/g).

### Determination of antioxidant activity

#### ABTS radical scavenging assay

For ABTS assay, the method of Re et al. [32] was adopted. The stock solutions included 7 mM ABTS solution and 2.4 mM potassium persulfate solution. The working solution was then prepared by mixing the two stock solutions in equal quantities and allowing them to react for 12 h at room temperature in the dark. The solution was then diluted by mixing 1 ml ABTS^.+ ^solution with 60 ml methanol to obtain an absorbance of 0.706 ± 0.001 units at 734 nm using the spectrophotometer. Fresh ABTS^.+ ^solution was prepared for each assay. Plant extracts (1 ml) were allowed to react with 1 ml of the ABTS^.+ ^solution and the absorbance was taken at 734 nm after 7 min using the spectrophotometer. The ABTS^.+ ^scavenging capacity of the extract was compared with that of BHT and percentage inhibition calculated as ABTS radical scavenging activity (%) = [(Abs_control _- Abs_sample_)]/(Abs_control)_] × 100 where Abs_control _is the absorbance of ABTS radical + methanol; Abs_sample _is the absorbance of ABTS radical + sample extract/standard.

#### DPPH radical scavenging assay

The effect of extracts on DPPH radical was estimated using the method of Liyana-Pathirana & Shahidi [33]. A solution of 0.135 mM DPPH in methanol was prepared and 1.0 ml of this solution was mixed with 1.0 ml of extract in methanol containing 0.02–0.1 mg of the extract. The reaction mixture was vortexed thoroughly and left in the dark at room temperature for 30 min. The absorbance of the mixture was measured spectrophotometrically at 517 nm. Ascorbic acid and BHT were used as references. The ability to scavenge DPPH radical was calculated by the following equation: DPPH radical scavenging activity (%) = [(Abs_control _- Abs_sample_)]/(Abs_control)_] × 100 where Abs_control _is the absorbance of DPPH radical + methanol; Abs_sample _is the absorbance of DPPH radical + sample extract/standard.

#### Total antioxidant activity (FRAP assay)

A modified method of Benzie & Strain [34] was adopted for the FRAP assay. The stock solutions included 300 mM acetate buffer (3.1 g C_2_H_3_NaO_2_·3H_2_O and 16 ml C_2_H_4_O_2_), pH 3.6, 10 mM TPTZ (2, 4, 6-tripyridyl-*s*-triazine) solution in 40 mM HCl, and 20 mM FeCl_3_·6H_2_O solution. The fresh working solution was prepared by mixing 25 ml acetate buffer, 2.5 ml TPTZ, and 2.5 ml FeCl_3_·6H_2_O. The temperature of the solution was raised to 37°C before using. Plant extracts (150 μL) were allowed to react with 2850 μL of the FRAP solution for 30 min in the dark condition. Readings of the colored product (ferrous tripyridyltriazine complex) were taken at 593 nm. The standard curve was linear between 200 and 1000 μM FeSO_4_. Results are expressed in μM Fe (II)/g dry mass and compared with that of BHT, ascorbic acid and catechin.

#### Bioassay

The bacterial cultures used in this study were obtained from the Department of Biochemistry and Microbiology, Rhodes University, South Africa. They consisted of five Gram-positive and five Gram- negative strains (Table 1). Each organism was maintained on nutrient agar plates and was recovered for testing by growth in nutrient broth for 24 hrs. Before use, each bacterial culture was diluted 1:100 with fresh sterile nutrient broth [35].

**Table 1:** Antibacterial activity of the leaves and stem extracts of *Buddleja saligna*

	Minimum inhibitory concentration (mg/ml)				
Bacterial species	Gram +/-	Leaves	Stem	Chloramphenicol μg/ml	Streptomycin μg/ml

*Bacillus cereus*	+	2.5	2.5	<2	<2

*Staphylococcus epidermidis*	+	5.0	na	<2	<2

*Staphylococcus aureus*	+	5.0	na	<2	<2

*Micrococcus kristinae*	+	5.0	na	<2	<2

*Streptococcus pyogens*	+	2.5	2.5	<2	<2

*Escherichia coli*	-	5.0	na	<2	<2

*Salmonella pooni*	-	5.0	na	<2	<2

*Serratia marcescens*	-	na	na	<2	<2

*Pseudomonas aeruginosa*	-	na	5.0	<2	<5

*Klebsiella pneumonae*	-	5.0	na	<2	<2

Test organisms were streaked in a radial pattern on sterile nutrient agar plates containing filtered extracts at final concentrations of 0.1, 0.5, 1.0, 2.5 and 5.0 mg/ml [25,36]. Plates containing only nutrient agar and another set containing nutrient agar and the respective solvents served as controls. After inoculation, the plates were incubated at 37°C for 24 to 48 hours. Each treatment was performed in triplicate and complete inhibition of bacterial growth was required for an extract to be declared bioactive.

### Statistical analysis

The experimental results were expressed as mean ± standard deviation (SD) of three replicates. Where applicable, the data were subjected to one way analysis of variance (ANOVA) and differences between samples were determined by Duncan's Multiple Range test using the Statistical Analysis System (SAS, 1999) program. *P *Values < 0.05 were regarded as significant.

## Results

### Total phenolic, flavonoids and proanthocyanidin contents

Results obtained in the present study revealed that the level of these phenolic compounds in the methanol extracts of the leaves and stem of *B. saligna *were considerable and as shown in Table 2. The total polyphenols and proanthocyanidins of the stem extract are higher than that of the leaves while the leave extract has higher level of flavonoids and total flavonols.

**Table 2:** Polyphenol contents of the methanol extracts of the leaves and stems of *Buddleja saligna* (n = 3, X ± SEM).

Phenolics	Leaves	Stems
Total polyphenol ^a^	15.65 ± 0.21	25.31 ± 1.37*

Flavonoids ^b^	1.61 ± 0.01	0.83 ± 0.02*

Total Flavonol ^c^	0.76 ± 0.05	0.10 ± 0.02*

Proanthocyanidins ^d^	1.67 ± 1.01	4.80 ± 0.35*

^a^Expressed as mg tannic acid/g of dry plant material.

^b^Expressed as mg quercetin/g of dry plant material.

^c^Expressed as mg quercetin/g of dry plant material.

^d^Expressed as mg quercetin/g of dry plant material

* indicates that this value is significantly different from the other at P < 0.05

### Total antioxidant power (FRAP)

The FRAP values for the methanol extract of the leaves of *B. saligna *were significantly lower than that of ascorbic acid and catechin, but higher than that of BHT. The methanol extract of the stem on the other hand has its FRAP values comparable to that of vitamin C but higher than that of catechin and BHT. The reducing ability of the extracts was in the range of 490.98 – 1546.98 μm Fe (II)/g. Antioxidant activity increased proportionally to the polyphenol content (Table 3).

**Table 3:** FRAP activity of the leaves and stem extracts of *Buddleja saligna*

Extracts	FRAP ^e^
Leaves	490.98 ± 38.89

Stem	1546.98 ± 63.67

Ascorbic acid	1632.1 ± 16.95

BHT	63.46 ± 2.49

Catechin	972.02 ± 0.61

Quercetin	3107.29+31.28

^e^Expressed in units of μmol Fe (II)/g.

### DPPH radical scavenging activity

**Figure 1:**
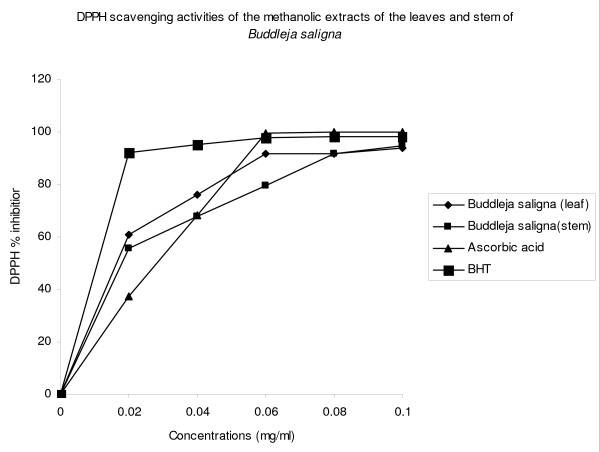
**DPPH scavenging activity of the methanolic extracts of the leaves and stem of *Buddleja saligna***.

Figure 1 shows the dose-response curve of DPPH radical scavenging activity of the methanol extracts of the leaves and stem of *B. saligna*, compared with BHT and ascorbic acid. It was observed that methanol extracts of the leaves and stem of *B. saligna *had similar activity. At a concentration of 0.1 mg/ml, the scavenging activity of methanol extract of the leaves reached 93.8%, while at the same concentration, that of the stem was 94.9%.

### ABTS radical scavenging activity

**Figure 2:**
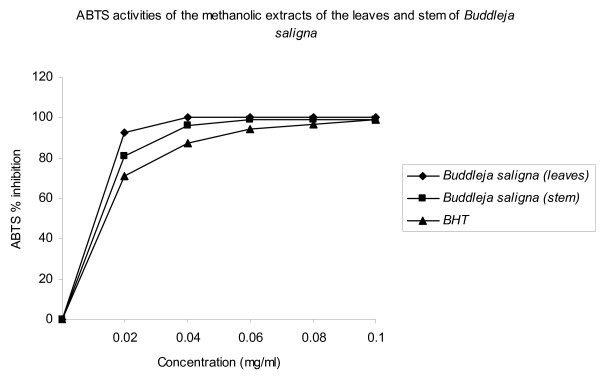
**ABTS activities of the methanolic extracts of the leaves and stem of *Buddleja saligna***.

The methanol extracts of the leaves and stem of *B. saligna *were fast and effective scavengers of the ABTS radical (Figure 2) and this activity was comparable to that of BHT. The percentage inhibition was 100, 98.8 and 99.3% for the leaf extract, stem extract and BHT respectively.

### Antibacterial activity

The antibacterial activity of the methanol extracts of the leaves of the *B. saligna *is much higher than of the stem. The methanol extract of the leaves of this plant has activity against all the organisms except *Serratia marcescens *and *Pseudomonas aeruginosa *and that at 2.5 and 5 mg/ml. The extract of the stem on the other hand was only active against *Bacillus cereus, Streptococcus pyrogens *and *Pseudomonas aeruginosa *at similar concentrations.

## Discussion

Polyphenols are the major plant compounds with antioxidant activity. This activity is believed to be mainly due to their redox properties [37], which play an important role in adsorbing and neutralizing free radicals, quenching singlet and triplet oxygen, or decomposing peroxides. The results strongly suggest that phenolics are important components of these plants, and some of their pharmacological effects could be attributed to the presence of these valuable constituents.

The antioxidant potentials of the methanol extracts of the leaves and stem of *B. saligna *were estimated from their ability to reduce TPRZ-Fe (III) complex to TPTZ-Fe (II). According to recent reports, a highly positive relationship between total phenols and antioxidant activity appears to be the trend in many plant species [38].

Proton radical scavenging is an important attribute of antioxidants. ABTS, a protonated radical, has characteristic absorbance maxima at 734 nm which decreases with the scavenging of the proton radicals [39]. Higher concentrations of the extracts were more effective in quenching free radicals in the system.

The effect of antioxidants on DPPH is thought to be due to their hydrogen donating ability [40]. The DPPH radical scavenging abilities of the extracts were significantly comparable to those of ascorbic acid (100%) and BHT (98.3), showing that the extracts have the proton-donating ability and could serve as free radical inhibitors or scavengers, acting possibly as primary antioxidants. The scavenging of the ABTS radical by the extracts was found to be slightly higher than that of DPPH radical. Factors like stereoselectivity of the radicals or the solubility of the extract in different testing systems have been reported to affect the capacity of extracts to react and quench different radicals [41]. Wang et al. [42] found that some compounds which have ABTS scavenging activity did not show DPPH scavenging activity.

The antibacterial activity of the extracts of the leaves and stem of the plant is presented in Table 1. It has just been shown that the plant extracts were active against the Gram-positive and some Gram-negative strains. This observation therefore supports the fact that, in general, the Gram-negative bacteria are more resistant than the Gram positive ones [43,44]. Since these extracts show some activity against all the organisms used in this study; the use of this plant for medicinal purpose is being justified.

## Conclusion

The results from this study indicate that the leaves and stem extracts of *Buddleja saligna *possess antioxidant properties and could serve as free radical inhibitors or scavenger or, acting possibly as primary antioxidants. Although, the antibacterial properties of *Buddleja saligna *are not as effective as the standard drugs-Chloramphenicol and Streptomycin, they still possess some activity against bacterial strains used in this study. *Buddleja saligna *may therefore be a good candidate for functional foods as well as pharmaceutical plant-based products.

## Competing interests

The authors declare that they have no competing interests.

## Authors' contributions

AAA carried out the assay, drafted the manuscript. FOJ prepared the extract and carried out assay. SV prepared the extract and carried out the assay. PJM coordinated the study and also provided the grant for the study. AJA coordinated the study. All authors read and approved the final manuscript.

## Pre-publication history

The pre-publication history for this paper can be accessed here:


